# Characterizing the face in facioscapulohumeral muscular dystrophy

**DOI:** 10.1007/s00415-020-10281-z

**Published:** 2020-10-28

**Authors:** T. G. J. Loonen, C. G. C. Horlings, S. C. C. Vincenten, C. H. G. Beurskens, S. Knuijt, G. W. A. M. Padberg, J. M. Statland, N. C. Voermans, T. J. J. Maal, B. G. M. van Engelen, K. Mul

**Affiliations:** 1 Department of Neurology, Donders Institute for Brain, Cognition and Behaviour, Radboud University Medical Center, PO Box 9101, 6500 HB Nijmegen, The Netherlands; 2 Radboudumc 3D-Lab, Department of Oral and Maxillofacial Surgery, Radboud University Nijmegen Medical Centre, Nijmegen, The Netherlands; 3 Department of Orthopedics, Section of Physical Therapy, Radboud University Medical Center, Nijmegen, The Netherlands; 4 Department of Rehabilitation, Donders Institute for Brain, Cognition and Behaviour, Radboud University Medical Center, Nijmegen, The Netherlands; 5 Department of Neurology, University of Kansas Medical Center, Kansas City, KS USA

**Keywords:** Facioscapulohumeral muscular dystrophy, Facial weakness, Outcome measures

## Abstract

**Objective:**

To evaluate facial weakness in patients with FSHD to better define clinical signs, and pilot a facial weakness severity score.

**Methods:**

87 FSHD patients and 55 controls were video recorded while performing seven facial tasks. The videos were assessed by three independent examiners to compile an overview of signs of facial weakness. Next, videos were semi-quantitatively assessed using a newly developed 4-point facial weakness score (FWS). This score was evaluated and correlated to other FSHD disease characteristics.

**Results:**

Patients had lower scores on the total FWS than controls (mean score 43 ± 28, range 4–118, vs 14 ± 9, range 0–35, *p* < 0.001) and on all seven individual facial tasks (all *p* < 0.001). 54% of patients had FWS scores outside the range of controls. Patients had more asymmetry between the left and right side of the face than controls. About 10% of the patients had very mild facial weakness. These were mostly males (89%) with longer D4Z4 repeat sizes of 7–9 units. More severe facial weakness correlated to more severe overall disease severity and shorter D4Z4 repeat size, but not to disease duration. Interobserver agreement for the FWS between three raters was low with a Fleiss Kappa of 0.437.

**Conclusion:**

This study provides an overview of the clinical spectrum of facial weakness and its relation to other disease characteristics. The 4-point scale we introduced to grade the severity of facial weakness enables correlation of facial weakness to disease characteristics, but is not suited as clinical outcome measure for longitudinal studies.

## Introduction

Facioscapulohumeral muscular dystrophy (FSHD) is a progressive inherited muscle disorder. A highly characteristic sign of FSHD is facial weakness that may vary between patients from minimal asymmetry to myopathic facies [1, 2]. The circular muscles around the eyes and mouth (orbicularis oculi and orbicularis oris, respectively) and the muscle that raises the corners of the mouth (zygomaticus major) are said to be commonly affected [1, 3]. A recent study suggests that other facial muscles, like for example the m. buccinator, may be affected in FSHD as well [2]. The facial weakness results in functional impairments such as difficulties in eating, drinking, speaking, and ocular problems, as well as in a reduced ability for facial expressions, hindering non-verbal communication.

In spite of its clinical relevance, facial weakness is a neglected feature in the consulting room and in research on FSHD [4]. Much is known about the specific pattern of involvement of the limb muscles, but studies describing the clinical characteristics of facial weakness in FSHD are scarce. Little is known about the prevalence and (variability in) the severity of facial weakness, its progression over time, relation to other disease characteristics, and the consequences of facial weakness for the patients.

This is probably due to the fact that we are lacking the clinical approach or tools to structurally assess facial weakness and measure changes, and the effect of those changes, over time.

To approach the current gap in the availability of tools for tracking facial weakness we video recorded faces of FSHD patients and controls to better define clinical signs of facial weakness to accommodate optimal examination. Next, we pilot a newly developed severity score for facial weakness to enable comparison between patients, correlations to other disease characteristics, and potentially track changes over time.

## Methods

### Patients

We included genetically confirmed FSHD patients aged 18 years or older from a large cross-sectional cohort study on FSHD performed at the Neurology department of the Radboud University Medical Center (Nijmegen, the Netherlands) from 2014 to 2015 (FSHD-FOCUS study) [5]. Study visit companions were invited to participate in the non-FSHD control group.

### Standard protocol approvals, registrations and patient consents

This study was conducted according to the principles of the Declaration of Helsinki (version October 2013) and in accordance with the Medical Research Involving Human Subjects Act (WMO). The study protocol was approved by the regional medical ethics committee (CMO Arnhem-Nijmegen). Prior to inclusion, all participants signed informed consent and if applicable a consent-to-disclose form.

### Data acquisition

**Fig. 1:**
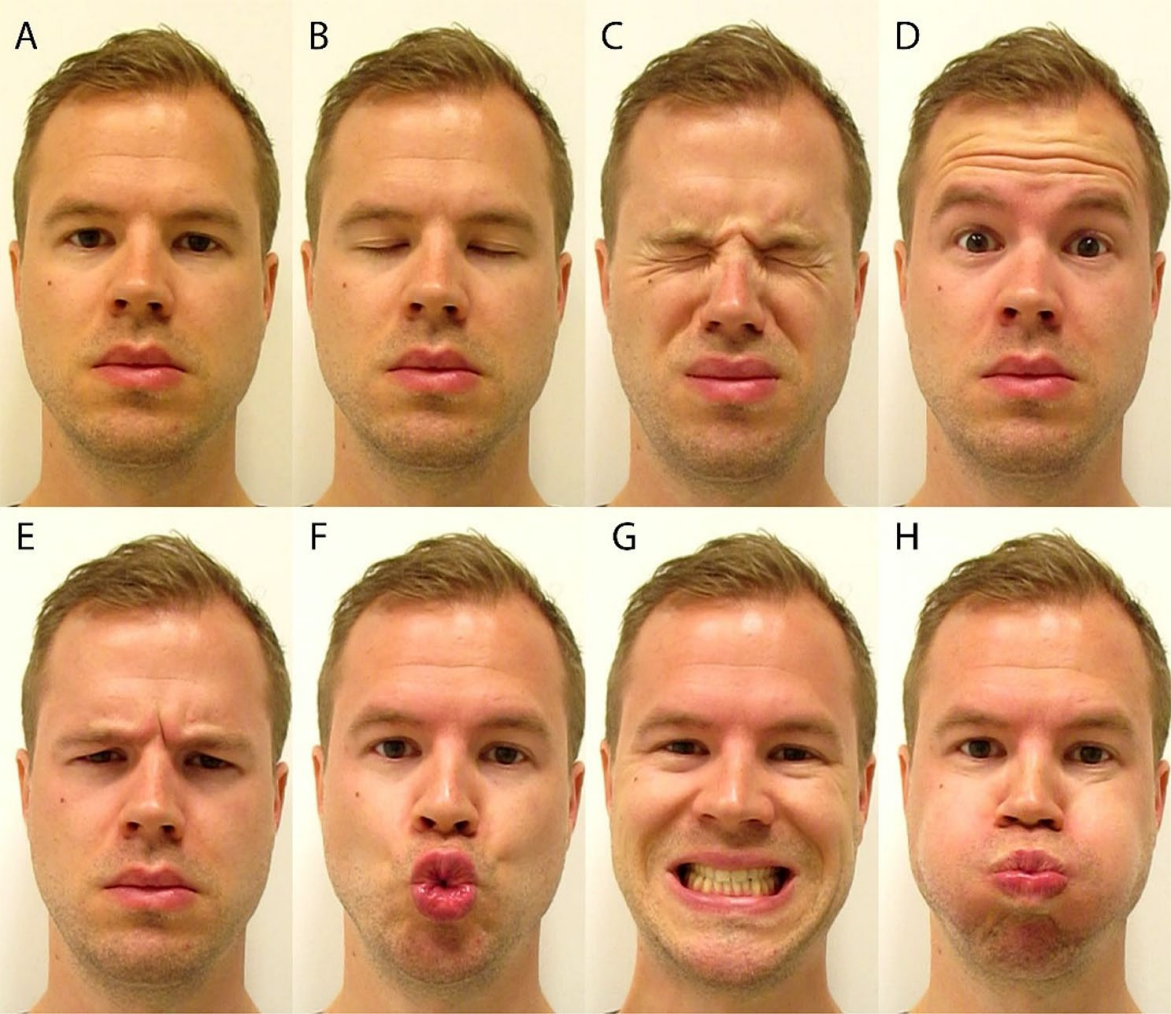
Face in rest and facial tasks in one of the controls. **a** Resting position; **b** closing the eyes gently; **c** closing the eyes firmly; **d** raising the eyebrows; **e** frowning; **f** pursing the lips; **g** showing the teeth and **h** puffing of the cheeks

All participants were video recorded while performing clinical exam procedures to elicit facial weakness. Seven different facial tasks were performed in random order: closing the eyes gently, closing the eyes firmly, raising the eyebrows, frowning, pursing the lips, showing the teeth and puffing of the cheeks (Fig. 1). All tasks started with a relaxed state of facial muscles and ended with the maximal movement possible. Each movement was performed three times in sequence during a session, resulting in twenty-one (seven times three) tasks per video (one video per participant).

The videos were recorded with a Canon PowerShot SX280 HS camera, which was positioned 70 cm in front of the participant. The camera was set to record at a resolution of 1920 × 1680 at 60 frames per second.

### Assessment of videos

The videos of all patients were independently assessed by three researchers (authors TL, SV and KM). Per video the researchers listed all clinical signs they noticed that could indicate facial weakness. Their assessments were combined to compile an overview of all signs noted to better define clinical signs of facial weakness.

Next, videos were semi-quantitatively assessed using a newly developed 4-point score for facial weakness, ranging from normal movements without effort (0) to the inability to initiate or perform a movement (3) (Table 1). Members of the study team with extensive experience with FSHD selected the set of facial movements likely to show changes in FSHD patients. Next, four different response options were formulated based on observations of clinical features in the videos. The assessments were performed independently by three experts in facial weakness from different disciplines [a mime therapist (CB), a neurologist specialized in FSHD (GP) and a speech language pathologist with special expertise in neuromuscular diseases (SK)]. All videos were cropped to an extend of which they only showed the complete face of participants. The videos were displayed to the experts in a randomized order, but the order of facial movements of the participants was fixed. Experts applied separate scores per task and for the left and the right side of the face as weakness is often asymmetrical. The experts were blinded for diagnoses of participants (FSHD patient or control). The total score of facial weakness was calculated by the sum of all scores (7 exercises bilaterally) of all three observers for one individual and further referred to as the ‘facial weakness score (FWS)’. As such, the total score ranged from 0 to 126, with higher scores indicating more severe weakness.

**Table 1:** Instructions on the scoring sheet for the facial weakness score

Score	Mouth and forehead	Eye closure
0	Complete movement possible	Complete eye closure
1	Near complete movement possible	Small rim of eyelashes visible on closure
2	Limited movement possible	Eyelashes mostly visible on closure
3	Impossible to initiate movement	Incomplete eye closure

### Clinical severity score

To assess overall disease severity the FSHD evaluation score and the clinical severity score by Ricci et al. (‘Ricci score’) were used. The FSHD evaluation score is a fifteen point scale which grades muscle weakness in six body regions with higher scores indicating more severe weakness [6]. The Ricci score is a ten point scale which grades overall disease severity with higher scores indicating more severe weakness [7].

### Facial sparing phenotype

To evaluate whether there were patients with a ‘facial sparing’ phenotype we identified patients with weakness of the limb muscles (FSHD evaluation score ≥ 6 or a Ricci score ≥ 3) whose facial weakness fell well within the range of the control group (within the lowest quartile of the FWS of the control group).

### Statistical analysis

Descriptive statistics were calculated for all participants. Mean and standard deviation are reported unless stated otherwise. The Fleiss Kappa value was calculated for the reliability of agreement between the three observers. Spearmen’s rho correlation was used for correlations between ordinal scores and Pearson correlation for linear variables. Statistical analysis were performed using SPSS version 22, version 3.5.3.

## Results

### Patient characteristics

Eighty-seven FSHD patients and fifty-five controls were included in this study. Baseline characteristics are presented in Table 2. Two of the eighty-seven FSHD patients received nocturnal non-invasive ventilation.

**Table 2:** Demographic data of study population

	Patients *n* = 87	Controls *n* = 55	*p* value
Male (*n*, (%))	51 (58%)	27 (47%)	> 0.05
Age in years (mean ± SD [range])	56.0 ± 15.4 [23–86]	50.9 ± 16.3 [21–87]	> 0.05
FSHD type 1 FSHD type 2 FSHD type 1 + 2	*n* = 80 *n* = 5 *n* = 2	n/a	
D4Z4 repeat array size in units 2–4 5–6 7–9 FSHD type 2	*n* = 15 *n* = 27 *n* = 40 *n* = 5	n/a	
Facial weakness score (mean ± SD [range])	43 ± 28 [4–118]	14 ± 9 [0–35]	< 0.001
FSHD evaluation score (mean ± SD [range])	6.9 ± 4.2 [0–15]	n/a	
Ricci score (mean ± SD [range])	5.5 ± 2.8 [0–10]	n/a	

*n/a* not applicable

### Overview of signs of facial weakness

**Fig. 2:**
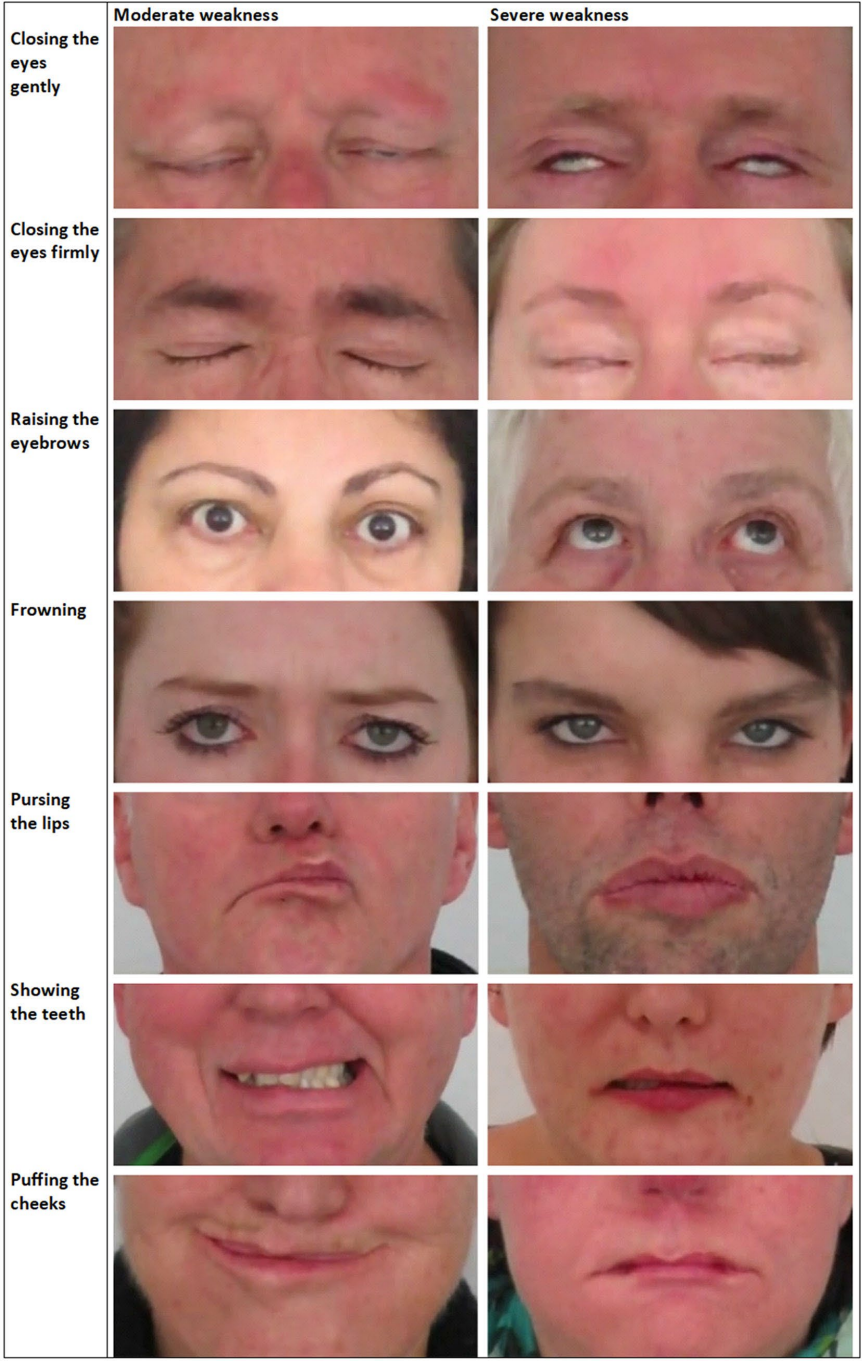
Examples of moderate and severe facial weakness per task (image taken of maximal movement possible)

An overview of all clinical signs of facial weakness that were noted in the video’s is given below to aid in the clinical examination of patients. Examples of moderate and severe weakness are displayed in Fig. 2.

#### Upper part of the face

In resting position, subtle weakness of the muscles around the eyes can be noted by a difference in palpebral fissure height, red conjunctiva when the eyes are dry, and in more severe cases hanging of the lower eyelid. Especially older patients may have less wrinkles around the eyes and on the forehead compared to controls of the same age.

When patients are asked to close their eyes (gently) a Bell’s phenomenon may occur, although this also occurred in some of the controls. In patients with more severe facial weakness there is an inability to fully close the eyes. By having the patient close the eyes forcefully more subtle weakness can be detected, especially a ‘signe de cils’— an inability to bury the eyelashes completely when attempting to close the eyes tightly. To compensate for the weakness around their eyes, FSHD patients are more likely to move their mouth when asked to close their eyes forcefully. Additionally, their foreheads show less wrinkling, as was also the case when asking the patients to raise their eyebrows or to frown.

In rest, the position of the eyebrows is asymmetrical in part of the patients. This becomes more evident when the patient raises the eyebrows or frowns. In contrast, in controls the asymmetry tends to resolve when the patient moves the eyebrows. The range of motion of the eyebrows is often smaller in FSHD patients compared to controls.

Although we did not specifically test the movement of the eyes, we did not see signs suggestive of involvement of extraocular muscles. No ptosis was seen.

#### Lower part of the face

In part of the patients the mouth is asymmetrical in rest which can be noticed by a difference in position of the corners of the mouth, or in more severe cases the philtrum is skewed to one side. Severe weakness of the muscles around the mouth can cause the lower lip to drop. Asymmetry becomes more pronounced when a patient performs a task like pursing the lips or showing the teeth (raising the corners of the mouth). When performing these tasks some patients are able to only partly execute the movement, or are able to complete the movement but not to maintain the final position.

When patients attempt to smile, a so called ‘transverse smile’ is often seen. Due to the inability to raise the corners of the mouth, the mouth moves horizontally which may look like a grin. In cases of very severe facial weakness patients are not able to raise the corners of the mouth at all and instead move their lower yaw forward when asked to show their teeth.

### Facial weakness score

**Fig. 3:**
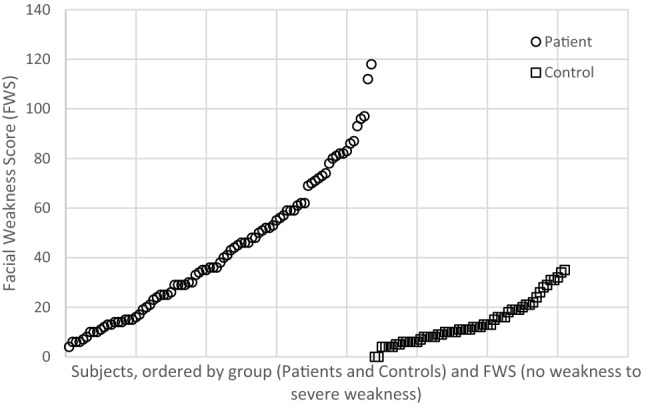
Facial weakness score per individual participant ranked per group from lowest to highest score

In patients the total score on the FWS ranged from 4 to 118, compared to a range between 0 and 35 in the controls. Although there was overlap between the scores of the patients and the controls, 54% of the patients had scores above the upper limit of the control scores (Fig. 3). Patients had lower scores than controls on the total FWS (mean score 43 ± 28 vs 14 ± 9 respectively, *p* < 0.001) as well as on all the seven individual facial tasks (all *p* < 0.001). There was no difference in the severity of the weakness between the upper and lower part of the face (no difference in mean scores for the tasks concerning weakness in the upper half versus the lower half of the face).

Males and females with FSHD had the same degree of facial weakness (mean total FWS 38 ± 25 vs 50 ± 30 respectively, *p* > 0.05).

Patients had more asymmetry between the left and the right side of the face than controls (mean absolute difference between scores for left and right side of the face 5.1 ± 3.6 vs 1.7 ± 1.8, *p* < 0.001). In patients, there was no preference for either right- or left-sided facial weakness.

**Fig. 4:**
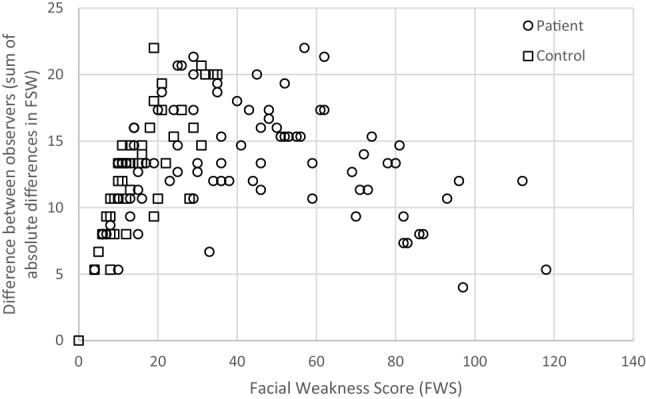
Sum of absolute differences in FWS between observers per individual. For individuals with moderate weakness the variability between observers is higher than for individuals with mild and severe weakness

Interobserver agreement for the total FWS score between the three experts was generally low with a Fleiss Kappa for the total FWS score of 0.437. Although the Fleiss kappa values varied somewhat between the various facial movement tasks, interobserver agreement was poor to moderate for all tasks (all Fleiss kappa ≤ 0.52). Collapsing the response categories from four to three (0–1–2–3 to 0–1–1–2) improved interobserver agreement to 0.498. Disagreement between the three experts was most pronounced with intermediate severity of facial weakness (Fig. 4).

### Correlation between FWS and FSHD disease characteristics

Facial weakness was more severe when overall disease severity was more severe on both the FSHD evaluation score and Ricci score (*ρ* 0.561, *p* < 0.001 and *ρ* 0.465, *p* < 0.001 respectively) and D4Z4 repeat size was shorter (*ρ* = − 0.507, *p* < 0.01).

Although patients with early onset FSHD (shoulder girdle weakness ≤ 10 years of age, *n* = 6) had more severe facial weakness than classical onset patients (mean FWS of 91 ± 17 vs 39 ± 25, *p* < 0.001), there was no correlation between disease duration and the degree of facial weakness (*r* = 0.204, *p* = 0.085). A weak correlation was found between the degree of facial weakness and age in the patients (*ρ* = 0.280, *p* = 0.009), but not in the control group (*ρ* = − 0.244, *p* = 0.072).

### Facial sparing phenotype

We identified nine out of 87 patients (eight males, age range 41–73 years) that fulfilled our criteria for a facial sparing phenotype. Their FWS results ranged from 7 to 15. All of these patients had a D4Z4 repeat array size of five to nine units, except for one who was an FSHD2 patient.

**Fig. 5:**
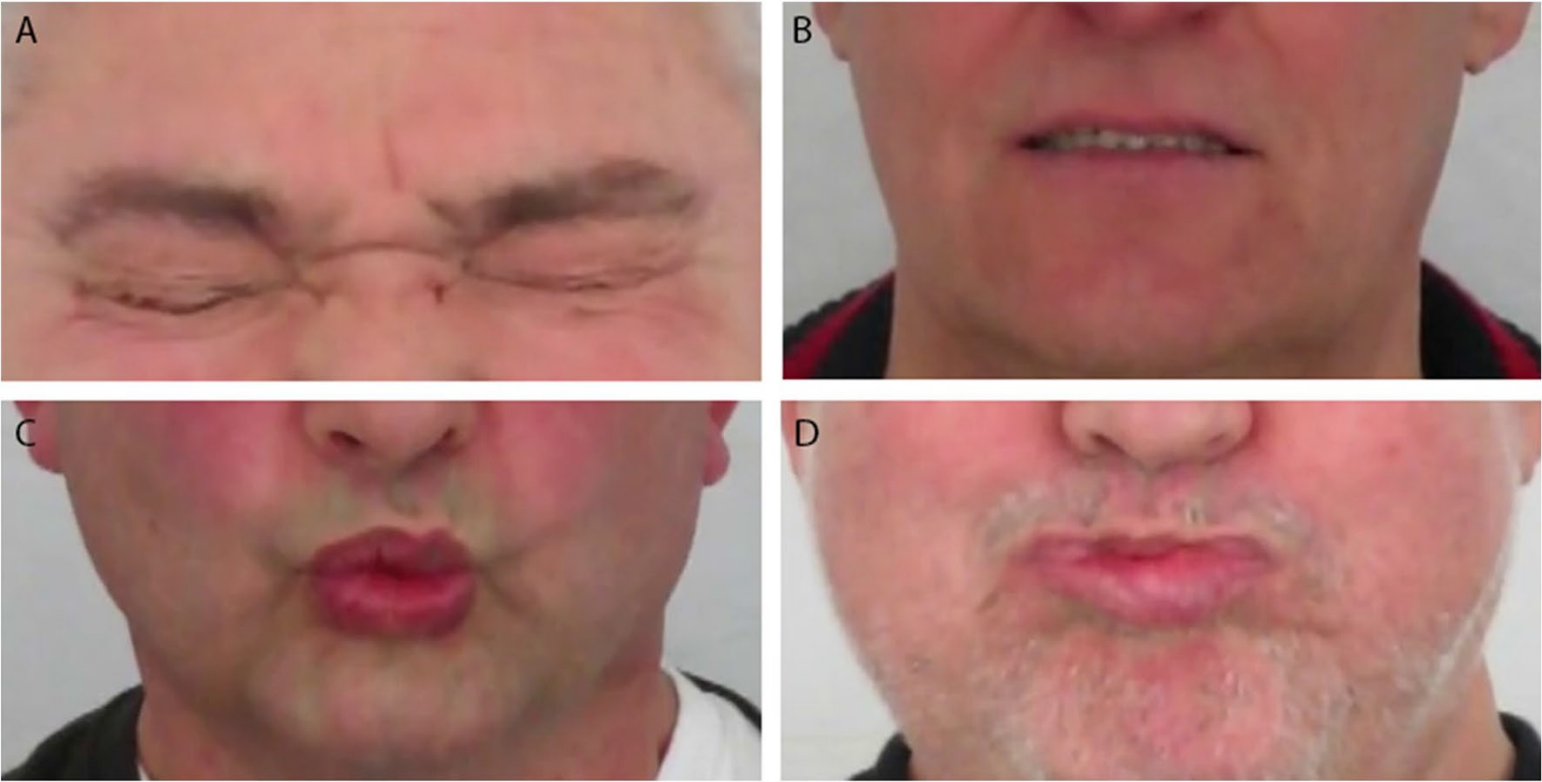
Examples of subtle signs of facial weakness. **a** Signe de cils right eye (inability to bury the eyelashes completely when attempting to close the eyes tightly). **b** Asymmetry in raising the corners of the mouth with left corner being raised less high. **c** Asymmetry when pouting the lips due to right sided weakness of the muscles around the mouth. **d** Difficulty puffing the cheeks. Notice the asymmetry and the lips being sealed horizontally

In six of these nine patients the experts observed clear, though mild, signs of facial weakness (all males, age range 48–73 years) (Fig. 5). All of these patients had notable asymmetry with movements of the mouth, i.e. single sided weakness of the muscles around the mouth. Three of them had signe de cils. One patient had trouble raising his left eyebrow.

The other three patients had such minimal signs of facial weakness that they could easily go unnoticed upon examination (2 males, ages 41, 47 and 62 years). One male patient only had rightsided signe de cils. The other male patient had signe de cils and although he was able to completely raise the corners of his mouth, he could not sustain the maximum position for more than a second with the left corner of his mouth. The female patient had asymmetrical movements of the corners of the mouth (right side performed suboptimal) when showing the teeth and blowing the cheeks.

## Discussion

In this study we systematically assessed facial weakness in FSHD in a large group of patients comprising the entire clinical spectrum.

Fifty-four percent of the FSHD patients had an FWS above the upper limit of the FWS of the control group. This indicates that the group of patients with moderate to severe weakness can adequately be distinguished from non-FSHD controls, but in the group of patients with milder weakness assessing facial weakness becomes more complex. Only two of all the participants had a completely normal score on the FWS. This finding shows that mild signs of facial weakness are not necessarily specific for FSHD, but can be seen in the general population.

One of the signs of facial weakness that is more specific for FSHD is left–right asymmetry, as asymmetry was more common in patients than controls and patients with very mild facial weakness mostly had single sided weakness. More research is required to define other signs of facial weakness that can help to reliably discriminate between patients and controls.

Some of the controls had relatively high scores (up to 35) on the FWS. These scores are probably the result of various factors influencing the score. First, despite verbal instructions some of the controls performed the movements technically poorly or not with maximum effort, which did not seem to be caused by muscle weakness. Second, the experts did not know if they were watching a video of a patient or a non-FSHD volunteer, which poses a risk of experimenter’s bias when scoring the tasks: a tendency of rating values to drift towards what is expected by the rater, i.e. a tendency to assign higher scores when they suspected that the participant had FSHD. This is based on the idea that experienced researchers evaluate the presence of FSHD in a holistic view of the face and movement of the face rather than based on individual parts and exercises. Finally, no information was collected regarding comorbidities that could have an effect on facial muscle function or facial expression in the control group.

Approximately 10% of the patients had very mild weakness (within the bottom 75% FWS range of the control group). This is line with previous studies reporting a facial sparing phenotype in approximately 15% of cases [3, 8]. Our study shows, however, that the definition of ‘facial sparing FSHD’ remains challenging. All patients in the study that could potentially be classified as having a facial sparing phenotype did have mild facial weakness, but this weakness fell within the range of what is seen in the control population. We identified multiple patients with very subtle signs of facial weakness, that were not recognized by the patient and could easily be overlooked by an examiner. As such, the term ‘facial sparing’ should be used with caution in the hands of an observer who is not thoroughly familiar with FSHD.

In accordance with the literature, very mild facial weakness was observed in patients with longer repeat array sizes of 7–10 D4Z4 units [8–13], which suits the finding that the D4Z4 repeat array has a stronger influence of the degree of facial weakness than on the upper and lower extremity muscle involvement [5].

Although there was no difference in mean FWS between males and females, we found that 89% of the patients with a facial sparing phenotype was male. This is in concordance with other studies reporting 67–100% of patients being male in series (≥ 3 patients) of patients with a facial sparing phenotype [8–10, 13–17]. Other sex differences have been observed in FSHD, such as a higher proportion of women among asymptomatic gene carriers and a higher frequency of STIR positive lesions on muscle MRI in males, but these are all still pathophysiologically unexplained.

We observed no correlation between the severity of the facial weakness and the duration of the disease, although there was a weak positive correlation between facial weakness and age in the patients. This suggests that facial weakness in FSHD shows only little progression over time compared to the limb muscles or that progression only occurs during a short time interval in life. Longitudinal studies should be performed to provide information on progression of facial weakness. However, to enable longitudinal studies, both in the setting of a clinical trial or natural history study, an adequate outcome measure for facial weakness is required.

The semi-quantitative ‘facial weakness score’ presented here allowed us to grade the severity of facial weakness and subsequently explore the relation between facial weakness and other disease characteristics. The score showed a poor agreement between different observers and a range of scores in the control group. Therefore, the proposed score in its current form serves to make progress in this research field, but cannot be used as a clinical outcome measure.

Different explanations for the low interrater agreement are possible. First, the experts who assigned the scores received limited training to perform the scoring and all three had a different background, which may result in different interpretations of findings on the facial tasks. Second, the risk of experimenter’s bias as mentioned above could result in different scores between observers, depending on whether they believed they were watching a video of a patient or a control. Third, properties of the score itself can contribute to a lower reliability. The number of response options may have been too high to be able to discriminate consistently between different answering options, although reducing the number of response categories only mildly improvement the interrater agreement, or the phrasing of the response options may have been unclear. Although unlikely to resolve the low interrater agreement, spontaneous mimical movements and assessment of the face at rest would be of interest to add in future work on this topic. Finally, the complexity of the face and its movements makes it challenging to develop a (semi)quantitative scoring system, which is also illustrated by the large intra- and interobserver variability when applying different scoring systems in studies focused on facial nerve dysfunction [18]. This complexity means that a scoring system must contain many parameters to provide a reliable measure. Finding an easy to implement measure for a complex problem seems a perfect challenge for a computer-controlled solution like artificial intelligence, thus more research is needed to investigate the feasibility of such a solution. Future development of a computer-controlled system will probably benefit longitudinal evaluation of facial weakness greatly.

This study provides an overview of the clinical spectrum of facial weakness and its relation to other disease characteristics. The task of the developing of an objective and accurate outcome measure for facial weakness is highly challenging and requires more research. The simple four point scale we introduced to grade the severity of facial weakness, enables correlation of facial weakness to disease characteristics, but is not suited as a clinical outcome measure for longitudinal studies.

## Data Availability

The data that support the findings of this study are available from the corresponding author upon reasonable request.
